# 

**Published:** 1892-11

**Authors:** 


### Society Meetings.

The Southern Surgical and Gynecological Association will hold its fifth annual meeting in Louisville, Ky., on Tuesday, Wednesday, and Thursday, November 15, 16, and 17, 1892, under the presidency of Dr. J. McFadden Gaston, of Atlanta. Mayor Tyler, of Louisville, has written a letter to the Chairman of the Committee of Arrangements, Dr. L. S. McMurtry, tendering the use of the Council Chamber to the Association for this meeting. The indications on all sides are such as to warrant the expectation of a large and enthusiastic assemblage. Members of the medical profession are most cordially invited to attend.

The New York State Association of Railway Surgeons will hold its second annual meeting in the Academy of Medicine, 17 West Forty-third street, New York City, Monday, November 14, 1892.

PROGRAMME.—MORNING SESSION, 9.30 A. M.

*Papers.*—1. Conservative Surgery as Applied to Railway Injuries. By Dr. R. S. Harnden, of Waverly. Discussion opened by Dr. L. S. Pilcher, of Brooklyn.

2. A Contribution to the Study of Amputations at the Hip-joint. By Dr. J. B. Murdock, of Pittsburg. Discussion opened by Dr. John A. Wyeth, of New York.

3. Osteogenesis and Osteoplasty in Crushing Lesions of the Extremities. By Dr. Thomas H. Manley, of New York. Discussion opened by Dr. Robert F. Weir, of New York.

Business meeting, 12.30 to 1.30.

AFTERNOON SESSION, 2.30 P. M.

4. President's Address.

5. Expert Examination and Testimony in Railway Cases. By Dr. B. A. Watson, of Jersey City. Discussion opened by Dr. Stephen Smith, of New York.

6. The Transportation of the Wounded upon Railways. By Dr. W. B. Outten, of St. Louis. Discussion opened by Dr. W. J. Galbraith, of Omaha.

7. Calendula as a Surgical Dressing. By Dr. A. Wilson Dods, of Fredonia.

The officers for 1892 are: President, George Chaffee, Brooklyn; Vice-Presidents, M. J. Baker, Hornellsville; P. A. Skiff, Frankfort; Secretary, M. Cavana, Oneida; Treasurer, A. M. Curtiss, Buffalo.

---

The Eleventh International Congress will meet in Rome, Italy, from September 24 to October 1, 1893. By an official letter, dated August 22, 1892, and signed by Professor Guido Baccelli, President, and Professor E. Maragliano, Secretary-General, Dr. A. Jacobi, of New York, has been directed to form an American sub-committee. Its membership is not yet complete, but on it are already found, beside that of the Chairman, the names of Drs. William Osler, of Baltimore; S. C. Busey, of Washington; N. S. Davis, of Chicago; Charles A. L. Reed, of Cincinnati; William Pepper, of Philadelphia; F. Peyre Porcher, of Charleston; James

Stewart, of Montreal; and Alexander J. C. Skene, of Brooklyn, N. Y. In the interest of facilitating the trip to Italy and reducing the expense, arrangements will be made with the steamship companies. According to a communication from the Central Committee—contained in a letter of the Secretary-General's, dated September 24th—the North German Lloyd proposes to reduce the fare to Genoa by twenty per cent., and that of the return trip by ten per cent. It is expected that still more favorable terms will be secured.

---

THE next meeting of the Buffalo Microscopical Club will be held in the lower lecture room of the Library Building, Monday evening, November 14, 1892, at 8.15 o'clock. The programme for the evening is as follows: Exhibition—Embryology of the Chick; Essay—The Use of the Microscope in the Diagnosis of Diseases of the Blood, by G. Carl Huber, M. D., F. R. M. S., Assistant Professor of Histology and Embryology, University of Michigan, Ann Arbor, Mich. All readers of the JOURNAL are cordially invited to be present.

### BOOKS RECEIVED.

Index-Catalogue of the Library of the Surgeon-General's Office, United States Army. Authors and subjects. Vol. XIII. Sialagogues-Sutugin. Washington : Government Printing Office. 1892.

A Manual of Jurisprudence and Toxicology. By Henry C. Chapman, M. D., Professor of Institutes of Medicine and Medical Jurisprudence, in the Jefferson Medical College of Philadelphia ; Member of the College of Physicians of Philadelphia, of the Academy of Natural Sciences of Philadelphia, of the American Philosophical Society, and of the Zoological Society of Philadelphia. With thirty-six illustrations, some of which are in colors. Philadelphia : W. B. Saunders, 913 Walnut street. 1892. Price, $1.25.

How We Treat Wounds To-Day. A Treatise on the Subject of Antiseptic Surgery, which can be understood by beginners. By Robert T. Morris, A. M., M. D., Instructor of Surgery at the New York Post-Graduate Medical School ; Late House Surgeon to Bellevue Hospital, New York ; Member Linnean Society of Natural History, New York ; Member New York County Medical Society ; Consulting Surgeon to the Woman's Hospital, Brooklyn. Fifth and revised edition. New York and London : G. P. Putnam's Sons. The Knickbocker Press. 1891.

Transactions of The Association of American Physicians. Seventh session, held at Washington, D. C., May 24, 25, and 26, 1892. Vol. VII. Philadelphia : Printed for the Association. 1892.

Gynecology. By G. W. Bratenahl, M. D., Assistant in Gynecology, Vanderbilt Clinic, New York, and Sinclair Tousey, M. D., Assistant Surgeon, Out-Patient Department, Roosevelt Hospital, New York. $1.00. Students' Quiz Series, No. 12.

Practice of Medicine. By Edwin T. Doubleday, M. D., Member of New York Pathological Society, and J. D. Nagel, M. D., Member of New York County Medical Association. $1.00. Students' Quiz Series, No. 6.

Tuberculosis of Bones and Joints. By N. Senn, M. D., Ph. D., Professor of Practice of Surgery in Rush Medical College ; Professor of Surgery in the Chicago Polyclinic ; Attending Surgeon Presbyterian

Hospital; Surgeon-in-Chief St. Joseph's Hospital; President of the American Surgical Association; President of the Association of Military Surgeons of the National Guard of the United States; Permanent Member of the German Congress of Surgeons, etc. Illustrated with 107 engravings (seven of them colored). In one handsome royal octavo volume. 520 pages. Extra cloth, $4.00 net; sheep, $5.00 net; half-Russia, $5.00 net. Philadelphia: The F. A. Davis Co., Publishers, 1231 Filbert street.

The Ready-Reference Handbook of Diseases of the Skin. By George Thomas Jackson, M. D., Chief of Clinic and Instructor in Dermatology, College of Physicians and Surgeons, New York; Professor of Dermatology in the Woman's Medical College of the New York Infirmary. In one handsome duodecimo volume of 534 pages, with 50 engravings and a colored plate. Cloth, $2.75. Philadelphia: Lea Brothers & Co. 1892.

Gonorrhea and Urethritis. By G. Frank Lydston, M. D., Professor of the Surgical Diseases of the Genito-Urinary System and Syphilology in the Chicago College of Physicians and Surgeons, etc., etc. Physicians' Leisure Library. Detroit, Mich.: George S. Davis. 1892. Paper, 25 cents; cloth, 50 cents.

A Manual of Medical Jurisprudence. By Alfred Swaine Taylor, M. D., F. R. S. Revised and edited by Thomas Stevenson, M. D., London, Fellow of the Royal College of Physicians, London; Lecturer on Medical Jurisprudence and Chemistry at Guy's Hospital; Examiner in Forensic Medicine in the University of London; External Examiner in Forensic Medicine in the Victoria University; Official Analyst to the Home Office. Eleventh American, edited with citations and additions from the twelfth English edition. By Clark Bell, Esq., President of the American International Medico-Legal Congress of 1893. Honorary Member of the Medico-Legal Society of France; of the Society of Mental Medicine of Belgium; Ex-President of the Medico-Legal Society of New York, etc., etc. *Quod hodie exemplis tuemur mox inter exempla erit.* Philadelphia: Lea Brothers & Co. 1892.

Memoranda of Poisons. By Thomas Hawkes Tanner, M. D., F. L. S. Seventh American, from the last London, edition. Revised by John J. Reese, M. D., Late Professor of Medical Jurisprudence and Toxicology in the University of Pennsylvania. Philadelphia: P. Blakiston, Son & Co. 1892.

Contributions of Physicians to the English and American Literature. By Robert C. Kenner, A. M., M. D. Detroit, Mich: George S. Davis. 1892.

Obstetrics. A Manual for Students and Practitioners. By Charles W. Hayt, M. D., House Physician, Nursery and Children's Hospital, New York. Being Volume II. of the Students' Quiz Series. Pocket size, 190 pages, $1.00. Philadelphia: Lea Brothers & Co. 1892.

---

Notice to Contributors.—We are glad to receive contributions from every one who knows anything of interest to the profession. Articles designed for publication in the Journal should be handed in before the first day of the month. The Editors are not responsible for the views or opinions of contributors. All communications should be addressed to the Managing Editor, 284 Franklin St., Buffalo, N. Y.

